# Population mortality during the outbreak of Severe Acute Respiratory Syndrome in Toronto

**DOI:** 10.1186/1471-2458-7-93

**Published:** 2007-05-29

**Authors:** Stephen W Hwang, Angela M Cheung, Rahim Moineddin, Chaim M Bell

**Affiliations:** 1 Centre for Research on Inner City Health, the Keenan Research Centre in the Li Ka Shing Knowledge Institute of St. Michael's Hospital; Department of Medicine, Division of General Internal Medicine, University of Toronto, Canada; 2 Women's Health Program, University Health Network; Department of Medicine, Division of General Internal Medicine, University of Toronto, Canada; 3 Department of Family and Community Medicine, University of Toronto, Canada; 4 The Keenan Research Centre in the Li Ka Shing Knowledge Institute of St. Michael's Hospital; Department of Medicine, Division of General Internal Medicine, University of Toronto, Canada

## Abstract

**Background:**

Extraordinary infection control measures limited access to medical care in the Greater Toronto Area during the 2003 Severe Acute Respiratory Syndrome (SARS) outbreak. The objective of this study was to determine if the period of these infection control measures was associated with changes in overall population mortality due to causes other than SARS.

**Methods:**

Observational study of death registry data, using Poisson regression and interrupted time-series analysis to examine all-cause mortality rates (excluding deaths due to SARS) before, during, and after the SARS outbreak. The population of Ontario was grouped into the Greater Toronto Area (N = 2.9 million) and the rest of Ontario (N = 9.3 million) based upon the level of restrictions on delivery of clinical services during the SARS outbreak.

**Results:**

There was no significant change in mortality in the Greater Toronto Area before, during, and after the period of the SARS outbreak in 2003 compared to the corresponding time periods in 2002 and 2001. The rate ratio for all-cause mortality during the SARS outbreak was 0.99 [95% Confidence Interval (CI) 0.93–1.06] compared to 2002 and 0.96 [95% CI 0.90–1.03] compared to 2001. An interrupted time series analysis found no significant change in mortality rates in the Greater Toronto Area associated with the period of the SARS outbreak.

**Conclusion:**

Limitations on access to medical services during the 2003 SARS outbreak in Toronto had no observable impact on short-term population mortality. Effects on morbidity and long-term mortality were not assessed. Efforts to contain future infectious disease outbreaks due to influenza or other agents must consider effects on access to essential health care services.

## Background

In March 2003, the health care system in Toronto, Canada, was confronted with an outbreak of Severe Acute Respiratory Syndrome (SARS), a highly contagious and severe atypical pneumonia. [1-3] The infection control measures employed in response to this crisis were extraordinary and unprecedented. Actions to control SARS in the Greater Toronto Area included the closure of four hospitals, the cancellation of all non-emergency surgical services, an almost complete curtailment of inter-hospital patient transfers, and the postponement of most hospital-based outpatient clinics. [4,5] Moreover, access to physicians, hospital laboratories, imaging studies, and other high-technology services was limited by infection control procedures. [5] The effect of these measures on access to health care was underscored by media reports of inappropriate delays for cancer therapy and the inability to perform urgent surgeries, sometimes resulting in death. [6-9]

The effects of widespread limitations of access to medical services have been studied from different perspectives. Studies of influenza outbreaks from the early 20^th ^century provide insight into healthcare restrictions and are applicable to the SARS experience because both are contagious respiratory illnesses with high mortality rates that are largely controlled through isolation procedures. Previous work has documented increased cardiovascular and other non-influenza related deaths during influenza outbreaks. [10-12] However, these increases were likely related to the effects of the infection itself rather than limited access to care. [13-17] Studies of diminished health care access due to withdrawal of medical services during physicians' strikes have documented significant alterations in health service utilization, intermediate outcomes, and various health-related processes of care. [18-20] However, no rigorous study has examined the effects of physician job action on overall population mortality. [21]

While there is little debate regarding the necessity of a concerted response to control the SARS outbreak, the delivery of health care to the general population was clearly reduced. Evidence suggests that in Toronto more essential services were less affected than services for low-acuity conditions. [5] An important question is whether sharply curtailed access to health care had an impact on population mortality due to causes other than SARS. This issue will be highly relevant in the event of a future infectious disease emergency, such as a recurrence of SARS, a highly virulent influenza epidemic, avian flu, the emergence of a new infectious organism, or a bioterrorist attack with an agent such as smallpox. We therefore sought to determine if the infection control measures undertaken during the 2003 SARS outbreak in Toronto were associated with a change in overall population mortality.

## Methods

### Chronology of the SARS outbreak in Toronto

In early March 2003, a Toronto resident returned from Hong Kong infected with SARS. She died at home but spread the virus to her family members, who initiated the outbreak when they were admitted to hospital. However, since our intent was to estimate the effect of the public health containment measures on the overall population, we defined the period of the SARS outbreak as beginning on March 27, 2003 (the 13^th ^week of the year). [4] On this date, the province of Ontario declared a state of emergency and issued special directives to all hospitals in the Greater Toronto Area (City of Toronto, York Region, Durham Region, Peel Region, Halton Region, and Simcoe County) instructing them to curtail inter-hospital transfers, elective surgery, and outpatient services. [22] As a result, health care utilization in the Greater Toronto Area decreased significantly. [5] Inpatient procedures such as abdominal aortic aneurysm repairs, cholecystectomies, and joint replacement surgeries decreased by 24–55%. Cardiac surgeries and percutaneous coronary interventions decreased by about 40%. Outpatient procedures such as breast biopsies, chemotherapy infusions, and vasectomies decreased by 16–93%. Outpatient diagnostic tests such as MRI and CT scans decreased by 34–51%. Outpatient physician visits decreased by 16% overall, and emergency department visits decreased by 25%. In contrast, medical care in the area of Ontario outside of the Greater Toronto Area had increased infection control measures but comparatively little restriction of clinical services. We defined the end of the SARS outbreak as July 2, 2003 (the 27^th ^week of the year), because this was the date that the World Health Organization removed Toronto from its list of SARS-affected areas.

### Death certificate data

Death certificate data for the years 2001, 2002, and 2003 were obtained from the provincial death registry. All deaths in Ontario are recorded in this registry. Data obtained from each death certificate included the decedent's date of birth, sex, place of death, and date of death. Because our goal was to examine the indirect effects of SARS through its impact on the health care system, we subtracted the 44 deaths in Ontario due to SARS from the death certificate database before performing further analyses.

### Census data

National census data from Statistics Canada were used to determine the age-specific population of the Greater Toronto Area and the rest of Ontario at the midpoint of 2001, 2002, and 2003. To adjust for population growth, we used linear interpolation to estimate age-specific population in each week of 2001, 2002, and 2003. The total population of the Greater Toronto Area at the midpoint of 2003 was 2.93 million, and the total population of the rest of Ontario at the midpoint of 2003 was 9.30 million.

### Mortality rates

We combined death certificate and census data to calculate weekly age-specific mortality rates in the Greater Toronto Area and the rest of Ontario. Because the beginning of the 2003 SARS outbreak (as defined for the purpose of this study) occurred 12 weeks after the beginning of the year, we compared mortality rates for the 12-week period before the beginning of the outbreak, the outbreak period, and the 12-week period after the end of the outbreak. Mortality rates were calculated for the corresponding weeks in 2001 and 2002. Rates were expressed as deaths per week per 100,000 population. The two pre-specified primary analyses were based on total mortality in males and females of all ages, and total mortality in males and females age 65 years and over. These rates were not age- or sex-standardized because the age and sex structure of the population did not change significantly during the study period.

### Statistical analyses

We used two statistical approaches, Poisson regression and interrupted time series analysis. Poisson regression was used to compare weekly mortality rates in 2003 with corresponding weekly mortality rates in 2001 and 2002. The dependent variable was the log of the rtio of the observed to expected mortality rates. Generalized estimation equations were used to adjust for possible serial correlations.

We used interrupted time-series analyses to test for effects of the SARS outbreak on mortality rates. [23,24] Time-series analysis consists of several techniques for modeling autocorrelation in temporally sequenced data. [25] The Holt-Winters forecasting method was used to predict the mortality rate per 100,000 person-years during and after the SARS outbreak. This method takes into account both time trends and seasonal fluctuations within timeseries data. The 95% confidence intervals for predicted mortality rates were generated using this method and then compared to observed mortality rates.

Our primary analysis tested for a pulse-function effect with a delay of four weeks in the onset of changes in mortality rates at the beginning and end of the SARS outbreak. The four week delay was chosen as a best estimate of the lag between the beginning of the SARS emergency and onset of overall health effects. Sensitivity analyses were performed by varying the lag period from two weeks to six weeks. All statistical analyses were performed using SAS version 8.2 (SAS Institute, Cary, N.C.). This study was approved by the Research Ethics Board of St. Michael's Hospital.

## Results

**Figure 1:**
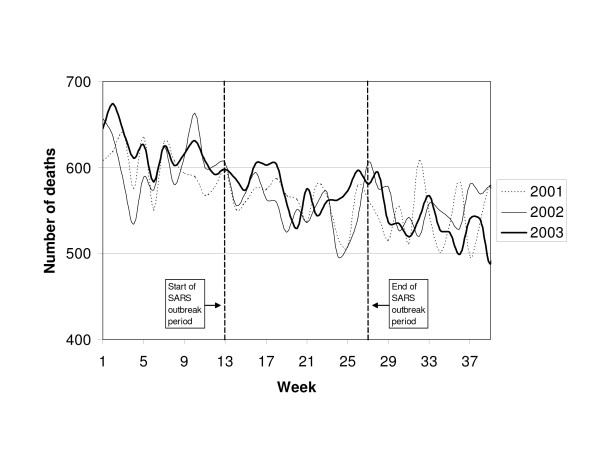
Weekly number of deaths in the Greater Toronto Area.

**Figure 2:**
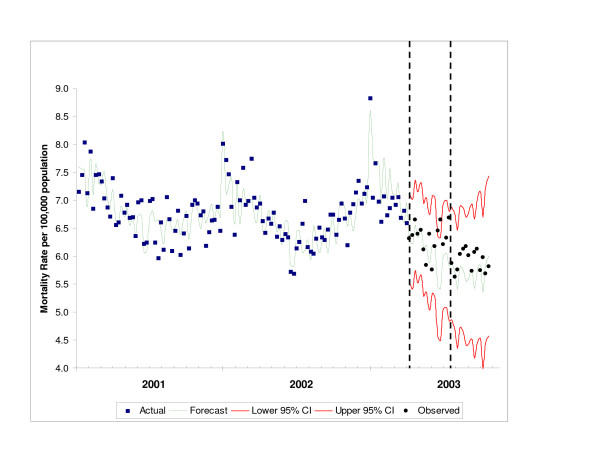
Weekly mortality rate per 100,000 population in the Greater Toronto Area. Dashed lines indicate the beginning and end of the SARS period. Observed mortality rates are indicated by squares and circles. Solid lines indicate the forecasted mortality rate and 95% confidence intervals.

During the period of the SARS outbreak between March 27 and July 2, 2003, there were 8,619 deaths in the Greater Toronto Area and 14,601 deaths in the rest of Ontario. The corresponding values were 8,350 and 14,677 in the year 2002, and 8,427 and 14,771 in the year 2001. The number of deaths in the Greater Toronto Area before, during, and after the 2003 SARS outbreak and the corresponding periods in 2001 and 2002 are shown in Figure 1. Figure 2 shows observed mortality rates in the Greater Toronto Area in 2003. The 95% confidence intervals are displayed for the expected mortality rate after the start of the SARS outbreak, based on the Holt-Winter method. The interrupted time series analysis found no significant change in mortality rates in the Greater Toronto Area associated with the period of the SARS outbreak in 2003, as indicated by the fact that the observed rates remained almost entirely within the 95% CI for the predicted rates.

### Adjusted analyses

The Poisson regression analysis found no significant change in mortality before, during, and after the period of the SARS outbreak in 2003 compared to the corresponding time periods in 2002 and 2001 (Table 1). The rate ratio for overall population mortality in the Greater Toronto Area was 0.99 [95% Confidence Interval (CI) 0.93–1.06] when mortality during the SARS outbreak in 2003 was compared to the corresponding time period in 2002. The rate ratio was 0.96 [95% CI 0.90–1.03] when mortality during the SARS outbreak in 2003 was compared to the corresponding time period in 2001. The rate ratio for overall population mortality in the rest of Ontario during the SARS outbreak was 0.97 [95% CI 0.91–1.02] when compared to 2002 and 0.97 [95% CI 0.92–1.02] when compared to 2001. The analysis of mortality rates among people aged 65 years and over also found no statistically significant changes (Table 1). A sensitivity analysis in which the lag period was varied from 2 weeks to 6 weeks yielded similar results.

**Table 1:** Poisson regression analyses of mortality rates.

	**Adjusted Mortality Rate Ratio (95% CI)**			
		2003 – Period Before SARS	2003 – Period During SARS	2003 – Period After SARS

				
**Greater Toronto Area**				
All ages	Compared to 2001	0.99 (0.91 – 1.08)	0.96 (0.90 – 1.03)	1.00 (0.92 – 1.10)
	Compared to 2002	1.05 (0.97 – 1.14)	0.99 (0.93 – 1.06)	1.01 (0.93 – 1.10)
Age 65 and over	Compared to 2001	0.99 (0.90 – 1.09)	0.97 (0.90 – 1.04)	1.00 (0.91 – 1.09)
	Compared to 2002	1.06 (0.97 – 1.17)	0.99 (0.92 – 1.07)	0.98 (0.90 – 1.07)
				
**Rest of Ontario**				
All ages	Compared to 2001	1.05 (0.99 – 1.12)	0.97 (0.92 – 1.02)	0.98 (0.93 – 1.04)
	Compared to 2002	1.07 (1.01 – 1.14)	0.97 (0.91 – 1.02)	0.97 (0.91 – 1.03)
Age 65 and over	Compared to 2001	1.05 (0.99 – 1.11)	0.94 (0.88 – 1.01)	0.95 (0.89 – 1.02)
	Compared to 2002	1.06 (1.00 – 1.13)	0.95 (0.89 – 1.02)	0.95 (0.89 – 1.02)

Adjusted mortality rate ratios compare mortality rates for periods in 2003 before, during, and after the SARS outbreak to the corresponding time periods in 2001 and 2002.

## Discussion

We examined overall mortality for the region most affected by the extraordinary infection control measures taken to contain the Toronto SARS outbreak in 2003. Despite a prolonged period of intense clinical service restrictions, we found no significant change in mortality rates compared with corresponding periods in previous years. The widespread limitation of access to medical services did not appear to have any short-term effects on deaths within the population.

Did the infection control measures and policies employed to control the SARS outbreak produce collateral damage to the delivery of health care to the population? The containment strategy did result in dramatic decreases in non-urgent inpatient and outpatient procedures and surgeries, outpatient diagnostic tests, and overall physician visits. [5] Although overall emergency department visits declined substantially during the SARS outbreak, this decline apparently reflected significant reductions in the volume of patients with low-acuity complaints. [5,26] Hospitalizations in Toronto during this period were 12% lower than expected, representing a relatively modest decrease. [27]

Studies conducted in other jurisdictions suggest that the changes in health care delivery and care-seeking behavior associated with the SARS outbreak may have had negative health effects. In Taiwan, use of ambulatory care fell by 24%, inpatient care decreased by 35%, and childbirths shifted from larger medical centers to less well-equipped district hospitals and clinics. [28,29] In Hong Kong, the number of individuals diagnosed with active tuberculosis decreased significantly during the outbreak, suggesting that the population's avoidance of medical care led to delays in diagnosis. [30] On the other hand, another study conducted in Hong Kong found that the proportion of positive samples for influenza and respiratory viruses other than SARS decreased significantly during the SARS outbreak, a change that the investigators attributed to the population's heightened attention to respiratory hygiene. [31] Because these acute viral infections can lead to serious complications among individuals with chronic medical conditions, this trend may have had a beneficial effect on the overall mortality rate.

Our data are consistent with the assertion that patients with severe illnesses retained the ability to access life-saving services during the SARS outbreak in Toronto. Fortunately, the 2003 outbreak was relatively short-lived and limited in scope. Future outbreaks of influenza or other emerging infectious diseases may be much more severe and prolonged. The establishment of data capture and reporting systems in advance of such an event would provide decision-makers with timely information on possible increases in morbidity and mortality attributable to impaired health care access, in addition to data on the direct effects of the outbreak itself. This information could be used to determine which services should be prioritized during a period of severely restricted health services.

This work has certain limitations. First, our study was designed to detect increases in short-term mortality that would constitute an immediate health challenge, but not longer-term effects. Although such delayed consequences are not implausible, they would be difficult to identify and attribute to impaired access to care during the SARS outbreak. Second, our study did not consider outcomes such as health status, quality of life, or other measures of morbidity that may have been affected by SARS-related infection control measures. Instead, we focused on mortality, an outcome that is both readily measured and of unquestioned importance. Future studies are needed to assess the effects of extraordinary infection control measures on morbidity. Third, we chose a clinically plausible lag of four weeks before the onset of change in mortality rates for our interrupted time-series analysis. The selection of a different lag period might have resulted in slightly different findings, although our results were corroborated by the Poisson regression analysis. Finally, this study examines a "natural experiment" in which restrictions on health care services were not applied uniformly, instantaneously, or in a randomized manner. Nonetheless, our use of a control group consisting of the population of Ontario outside the Greater Toronto Area, which was not affected by severe restrictions on access to hospital-based care, serves to minimize this potential bias.

## Conclusion

A coordinated response to severe infectious disease outbreaks requires an approach that balances an infection control mandate with the need to preserve access to essential health services. In the situation of a widespread disease outbreak, health care decision-makers understandably concentrate upon the immediate threat of the infectious disease. The SARS experience in Toronto suggests that the preservation of the delivery of health care for other urgent conditions is equally important. This concept is particularly relevant because future infectious disease outbreaks, such as avian influenza or a bioterrorist attack, could limit access to health care resources to a much greater extent than did the SARS outbreak. Further research is needed to examine the possible effects of such events on cause-specific mortality rates and on health outcomes other than mortality.

## Competing interests

The author(s) declare that they have no competing interests.

## Authors' contributions

SWH conceived of the study, participated in its design and coordination, and drafted the manuscript. AMC conceived of the study, participated in its design, and participated in the revision of the manuscript. RM performed the statistical analyses and participated in the revision of the manuscript. CMB conceived of the study, participated in its design and coordination, and drafted the manuscript. All authors read and approved the final manuscript.

## Pre-publication history

The pre-publication history for this paper can be accessed here:


